# Angioleiomyoma of the small intestine – a rare cause of gastrointestinal bleeding

**DOI:** 10.1186/1477-7819-5-129

**Published:** 2007-11-08

**Authors:** Umar Sadat, NS Theivacumar, Joe Vat, Asif Jah

**Affiliations:** 1 Cambridge Vascular Unit, Addenbrooke's Hospital, Cambridge University Hospitals NHS Foundation Trust, Cambridge, UK; 2 University Department of Surgery, Cambridge University Hospitals NHS Foundation Trust, Cambridge, UK; 3 University Department of Radiology, Cambridge University Hospitals NHS Foundation Trust, Cambridge, UK

## Abstract

**Background:**

Benign tumors are a rare cause of gastrointestinal hemorrhage of which angioleiomyomas constitute a very small minority. They have been reported in literature to present with volvulus, bleeding or intussusceptions.

**Case presentation:**

An interesting case of a patient presenting with gastrointestinal bleeding from an underlying angioleiomyoma is discussed along with its management options.

**Conclusion:**

Angioleiomyoma though rare can be managed successfully by surgical and/or minimally invasive endovascular procedures.

## Background

Angioleiomyoma of the small bowel is an extremely rare benign tumor. This is the first reported case in English literature, where a patient presenting with gastrointestinal bleeding had an underlying angioleiomyoma.

## Case presentation

A 58-year-old woman presented with a one-day history of malena and light-headedness. She did not have any known co morbidities. Apart from being anaemic she was haemodynamically stable and her general physical and abdominal examination unremarkable. Hematological examination revealed low hemoglobin levels of 7.3 g/dl with normal clotting profile. After initial resuscitation, an esophagogastroduodenoscopy (OGD) was performed which excluded any cause of upper gastrointestinal bleed. This was followed by colonoscopy, which although did confirm the presence of blood within the gastrointestinal tract but did not localize the site of bleeding. However, the patient's hemoglobin level continued to fall over the next two days, which was treated with a transfusion of six units of packed red cell.

**Figure 1:**
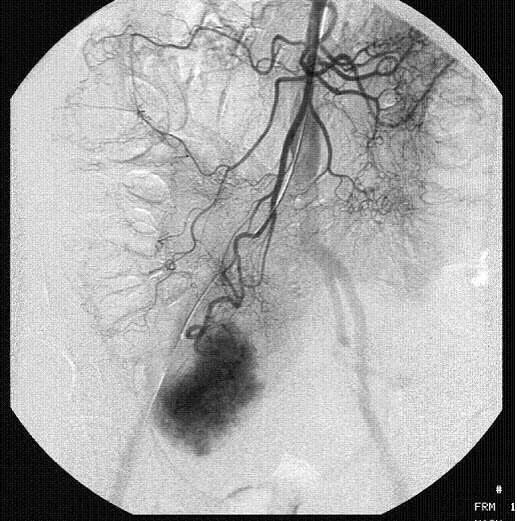
Selective superior mesenteric artery angiogram showing large feeding artery to a mass in the right iliac fossa with extensive vascular blush.

**Figure 2:**
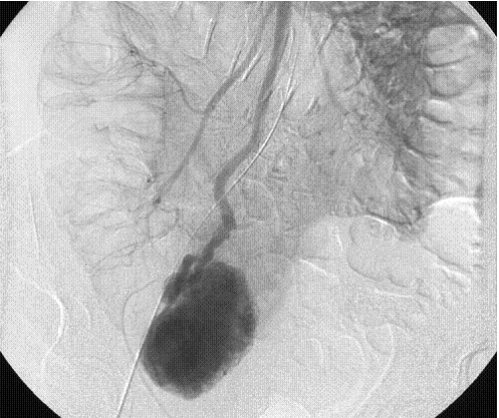
Venous phase of selective superior mesenteric artery angiogram showing large draining vein from the mass.

**Figure 3:**
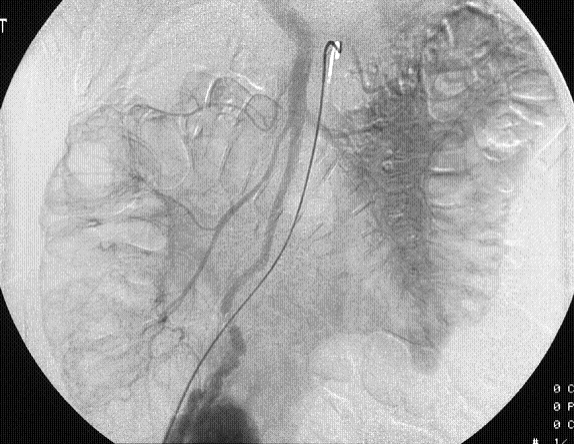
Communication of the draining vein into the portal venous system.

Subsequently, she underwent selective superior mesenteric angiography, which demonstrated a large hyper vascular mass with an extensive contrast blush, near the terminal ileum in the right iliac fossa (Figure 1). It was supplied by a large feeding artery and drained via a solitary large vein into the portal venous system (Figure 2, 3). No extravasation of contrast was seen suggesting that the lesion was not actively bleeding at the time of the study. Inferior mesenteric and coeliac axis angiography revealed no synchronous lesion or evidence of any vascular deposit in the liver.

**Figure 4:**
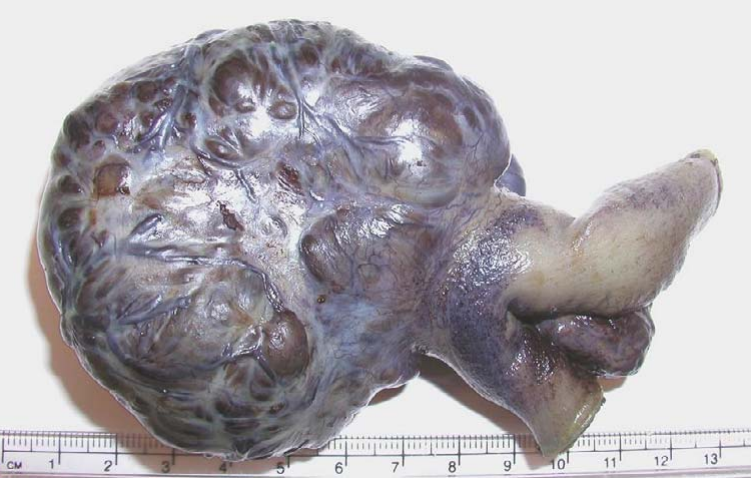
Excised tumor, measuring approximately 70 × 65 × 50 mm.

She thereafter underwent laparotomy through midline incision, which identified vascular solid tumor along the anti-mesenteric border of the ileum about 30 cm from the ileo-caecal junction, measuring 7 × 7 cm (Figure 4). A segmental small bowel resection was performed, and intestinal continuity established with an end-to-end small bowel anastomosis. The patient had an unremarkable postoperative recovery; her hematocrit remained stable and she was discharged from the hospital on the 7^th ^postoperative day.

**Figure 5:**
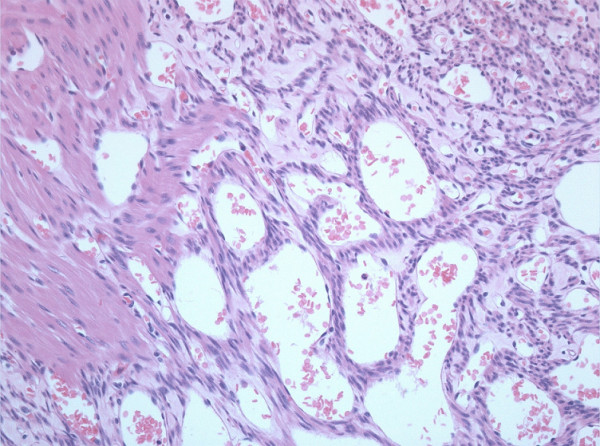
Histology of the angioleiomyoma with H&E staining.

The histopathology of the resected tumor showed a submucosal tumor compressing the mucosal lining causing ulceration. Sectional analysis of the tumour showed thick bands of muscle with vascular spongy stroma. Microscopic examination (Figure 5) showed thick bands of smooth muscle staining positive for smooth muscle antigens using immunochemistry. Immunochemistry was negative for gastrointestinal stromal tumors (C-Kit antigens) and sarcomas. Interspersed between these muscle fibers were a variety of vascular channels ranging in size from dilated capillaries to a small and medium sized arteries and veins (Figure 4). The smooth muscle appeared to arise from the muscularis propria of the small bowel. There was no significant mitotic activity in the tumor and no evidence of tumor necrosis. The tumor was well circumscribed with clear excision margins. All these findings were consistent with the diagnosis of angioleiomyoma.

## Discussion

Benign tumors are a rare cause of gastrointestinal hemorrhage, being responsible for fewer than 1% of all cases [1] and can present with conditions like volvulus, bleeding, obstruction and/or intussusceptions. Leiomyomas constitute a fraction of these benign lesions. They tend to occur mostly in the jejunum (44%) followed by ileum (37%) and duodenum (19%). Ezinger [2] distinguished, clinically and pathologically, three main groups of leiomyomas, namely: superficial leiomyomas, vascular leiomyomas or angioleiomyomas and deep leiomyomas.

Vascular leiomyomas are well-defined benign smooth muscle tumors with prominent abnormal thick walled venous channels [2]. The presence of vascular leiomyomas in the gastrointestinal tract is extremely rare but a few cases have been reported where they presented as volvulus [3], peritonitis [4] and perforation of the intestinal tract [5]. To the best of our knowledge this is the first English language case report where angioleiomyoma has presented primarily with intestinal bleeding. As the investigation for GI bleeding normally involves OGD and/or colonoscopy, these lesions are difficult to localize and this was our experience. Cross sectional imaging with CT or MRI can be used to detect mass lesions and delineate the anatomical extent. Mesenteric angiography is thus the investigation of choice in cases where OGD and colonoscopy do not reveal the source of bleeding. However definitive diagnosis is histological.

Four histological subtypes of angioleiomyomas [6,7] have been described namely:

• Capillary or solid angioleiomyomas having a rich smooth muscle cell stratification surrounding and holding a few thin vascular channels,

• Venous angioleiomyomas characterized by more numerous and thicker vascular channels than found in capillary angioleiomyomas,

• Cavernous angioleiomyomas having widened vascular channels surrounded by a thin layer of smooth muscle cells,

• Combined capillary and venous angioleiomyomas

Our patient had a mixed vascular angioleiomyomas with positive immunohistochemical staining with Desmin for smooth muscle cells. However, staining with Desmin is not necessary for such tumors as suggested by Hasegawa [8], Lundgren [9] and Matsuyama [10]. This characteristic is attributable to the presence of aberrant smooth muscle cells. According to Matsuyama and colleagues, expression of desmin in vascular smooth muscle cells also varies according to the anatomical site, the layer in the vascular wall, the kind or the size of the blood vessel, and the cellular condition such as in contractile or synthetic states [11-13].

One peculiar feature of the tumor found in our patient was its large size measuring 7 × 7 cm. Vascular leiomyomas are usually < 2 cm [14] although in exceptional cases they can reach large sizes because they are painless and therefore only diagnosed at a late stage.

The operative management is usually surgical involving resection of the affected bowel segment with end-to-end anastomosis as was done in our patient. Embolization of the feeding vessel can be performed as suggested by Cho and colleagues [15] to control exsanguination.

## Conclusion

Angioleiomyoma though rare can be managed successfully by surgical and/or minimally invasive endovascular procedures.

## Competing interests

The author(s) declare that they have no competing interests.

## Authors' contributions

**US **has been involved in the conception and design of the case report **ST**, **JV**, **AJ**, have been involved in the acquisition of data, analysis and interpretation of data, drafting the manuscript, revising it critically for important intellectual content. All authors read and approved final manuscript.
